# Supplementary material for: Anti-Ischemic Effects of PIK3IP1 Are Mediated through Its Interactions with the ETA-PI3Kγ-AKT Axis
Source: Cells. 2022 Jul 11;11(14):2162. doi: 10.3390/cells11142162 (PMC9322903; doi:10.3390/cells11142162)

Supplementary Table S1. List of antibodies for the present study.

Antibody	Purchase from	Cat#
Anti- HIF-1α	Cell signaling Technology	#14179
Anti- ET-1	abcam	ab2786
Anti- p-Akt (Ser473)	Cell signaling Technology	#4060
Anti- AKT	Cell signaling Technology	#9272
Anti- PIK3IP1	Santa Cruz	sc-365777
Anti- p53	Cell signaling Technology	#2527
Anti- BAX	Cell signaling Technology	#2774
Anti- Bcl-2	Cell signaling Technology	#3498
Anti- LC3B	Cell signaling Technology	#2775
Anti- c-Caspase-3 (Asp175)	Cell signaling Technology	#9661
Anti- PI3Kα	Santa Cruz	sc-293172
Anti- PI3Kγ	Santa Cruz	sc-166365
Anti- ETAR	abcam	ab178454
Anti- ETBR	abcam	ab117529
Anti- eNOS	Cell signaling Technology	#9572
Anti- PKG1	Cell signaling Technology	#3248
Anti- p-VASP (Ser239)	Cell signaling Technology	#3114
Anti- p-p44/42 MAPK (ERK1/2)(Thr202/Tyr204)	Cell signaling Technology	#9101
Anti- p44/42 MAPK (Erk1/2)	Cell signaling Technology	#9102
Anti- p-p70 S6 Kinase (Thr389)	Cell signaling Technology	#9205
Anti- p70 S6 Kinase	Cell signaling Technology	#9202
Anti- p-SAPK/JNK (Thr183/Tyr185)	Cell signaling Technology	#9251
Anti- SAPK/JNK	Cell signaling Technology	#9252
Anti- p-p38 MAPK (Thr180/Tyr182)	Cell signaling Technology	#9211
Anti- p38 MAPK	Cell signaling Technology	#9212

Supplementary Table S2. List of primer sets for the present study.

Gene	Forward primer (5'-3')	Reverse primer (5'-3')
18S	TTCTGGCCAACGGTCTAGACAAC	CCAGTGGTCTTGGTGTGCTGA
PIK3IP1	TTAGGATCCCATTTGGACACTGGCTG	GATCCTAGGCCTGTACCAGTGTTTAC
SOD1	GCAGAAGGCAAGCGGTGAAC	TAGCAGGACAGCAGATGAGT
SOD2	CTGAGGAGAGCAGCGGTCGT	CTTGGCCAGCGCCTCGTGGT
GPX1	CGATGCCACTGCCCTCAT	GGCCCACCAGGAACTTCTC
GPX4	GGCTACAATGTCAGGTT	TTATCAATGAGAAACTTGGTAA

Supplementary Figure S1

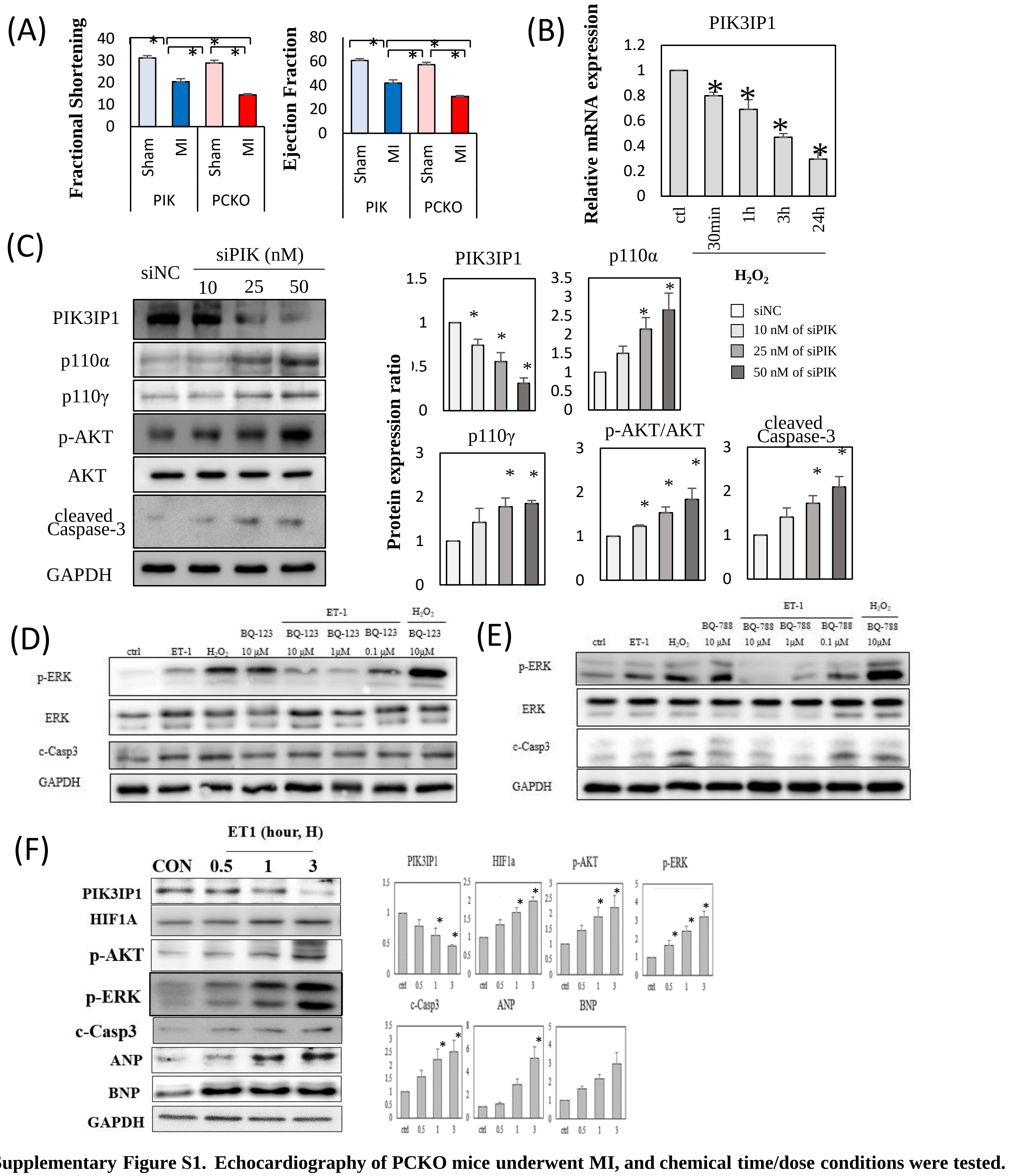

Supplementary Figure S1. Echocardiography of PCKO mice underwent MI, and chemical time/dose conditions were tested. (A) 8-weeks-old PIK3IP1fl/fl(PIK-control) and aMHC-Cre-PIK3IP1fl/fl(PCKO-knockout) mice underwent coronary artery ligation surgery. Fractional shortening and ejection fraction were measured by echocardiography at 6 weeks after myocardial infarction surgery. Note that knockout of PIK3IP1 worsened MI-induced cardiac injury. (B) Relative mRNA expression of PIK3IP1was measured by qRT-PCR with the PIK3IP1 primers (forward primer, 5′-TTAGGATCC CATTTGGACACTGGCTG-3′; reverse primer, 5′-GATCCTAGGCCTGTACCAGTGTTTAC-3′). The expression of mRNAs was normalized to 18S rRNA. All reactions were performed in triplicate. (C) H9c2 cells were transfected with siPIK (si-PIK3IP1) or 25 nM of siNC (negative control) for 24 h (10, 25, 50 nM each). (D,E) H9c2 cells were treated with/without ET-1 (10 nM) & H_2O_2 (200 μM) for 3h after pretreatment with BQ-123 or BQ-788 (0.1-10 μM). (F) H9c2 cells were transfected with 10 nM ET-1 for indicated times. The protein levels were measured by western blotting. The results were displayed in bar charts with means ± SEM; N=6; Statistical significance is shown as *P <0.05 relative to control (ctl) or siNC group.

Supplementary Figure S2

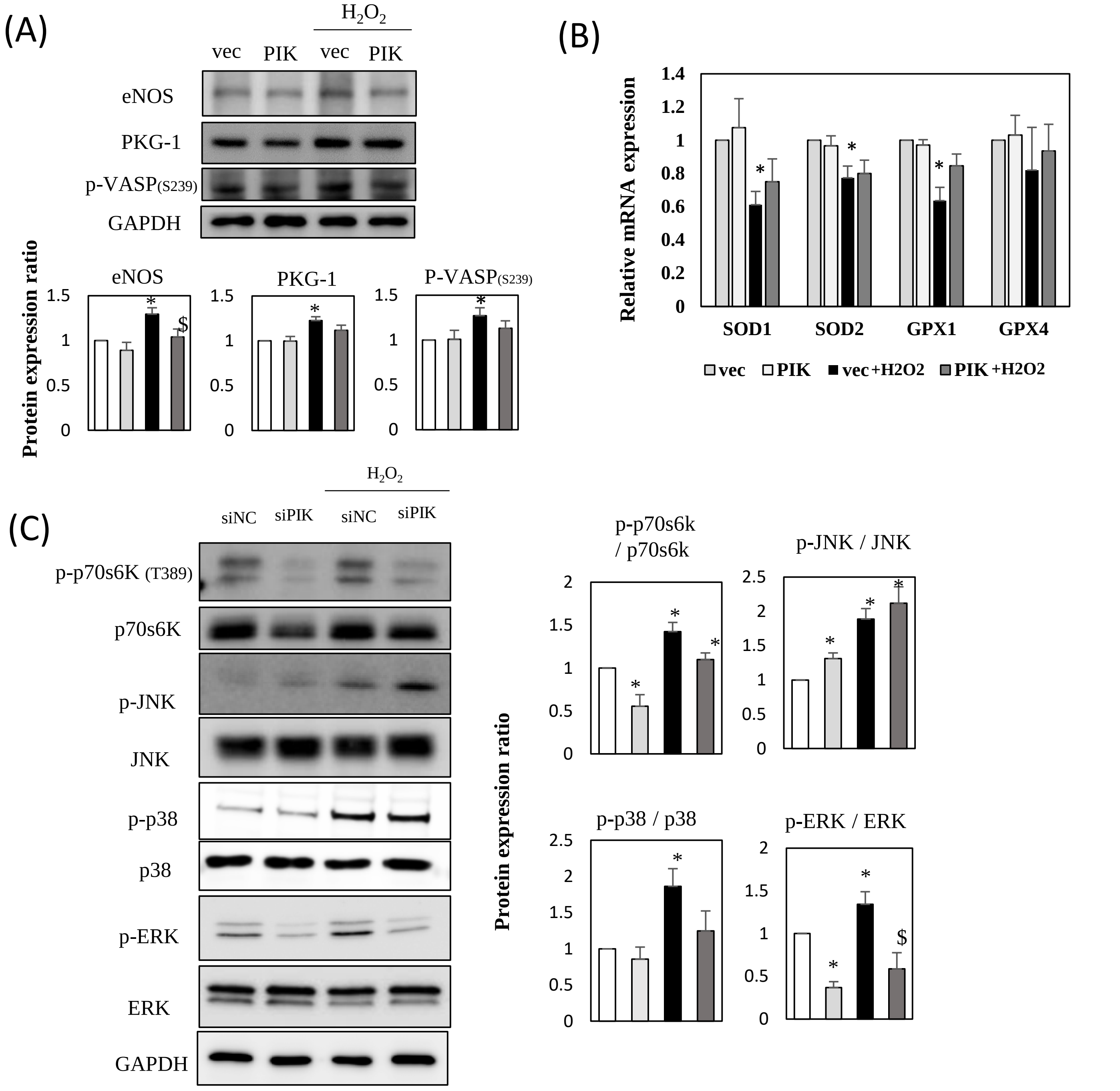

Supplementary Figure S2. **Regulation of PIK3IP1 is related with eNOS-PKG-p-VASP signaling, antidoxidant enzyme expression and MAPK signaling.** (A) H9c2 cardiomyocytes were transfected with pcDNA3.1 (Vec) or pcDNA3.1-PIK3IP1 (PIK) for 24h, and subsequently treated with or without H_2O_2 (200 μM). Expression levels of eNOS, PKG-1 and p-VASP were measured by western blotting and normalized to GAPDH. (B) Relative mRNA expressions of SOD1,SOD2, GPX1, and GPX4 were measured by qRT-PCR. The expression levels of the mRNAs were normalized to 18S rRNA. (C) H9c2 cells were transfected with siPIK (si-PIK3IP1) or siNC (negative control) for 24 h (25 nM each), and subsequently treated with or without H_2O_2 (200 μM). The protein levels of p-p70s6K, p-JNK, p-p38 and p-ERK were measured by western blotting. Each expression of phosphorylated form was normalized by the expression of p70s6K, JNK, p38 and p-ERK. The results for data displayed in bar charts are presented as means ± SEM; n=3; Statistical significance is shown as *P <0.05 relative to Vec or siNC group. $P <0.05 relative to siNC treated with H_2O_2.

Supplementary Figure S3

Uncropped gel images for **Figure 1C**

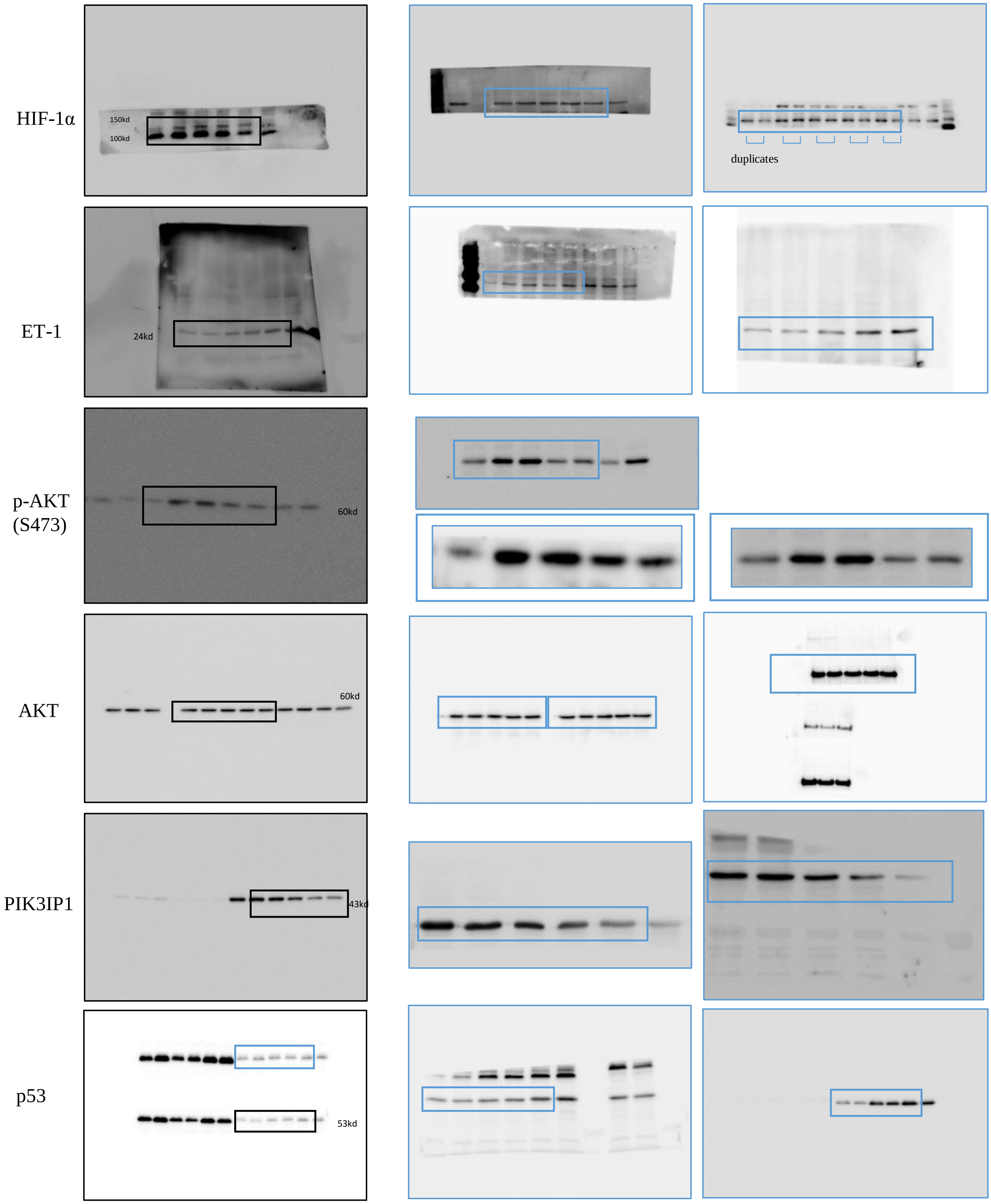

Supplementary Figure S3. Blots are for Figure 1C. Blots marked with black boxes are on the main figures and blots marked with blue boxes are replicates.

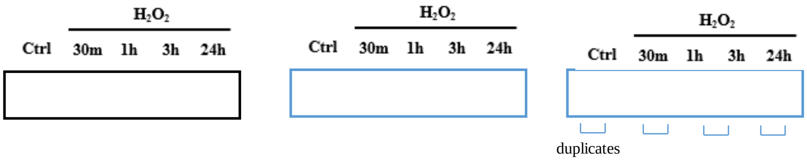

Supplementary Figure S4

Uncropped gel images for **Figure 1C**

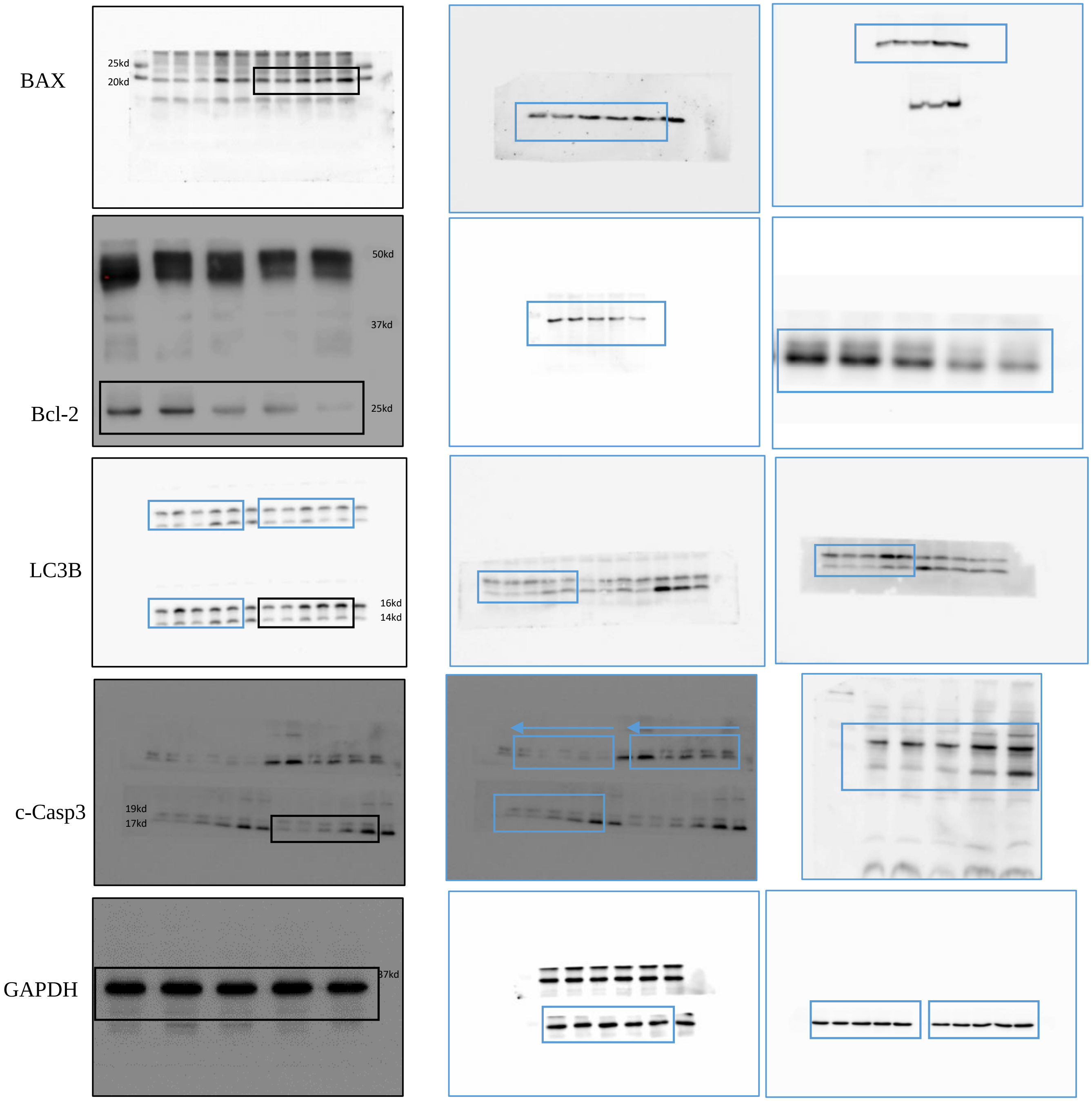

Supplementary Figure S4. Blots are for Figure 1C. Blots marked with black boxes are on the main figures and blots marked with blue boxes are replicates.

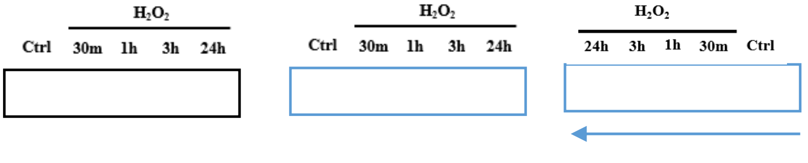

Supplementary Figure S5

Uncropped gel images for **Figure 2A**

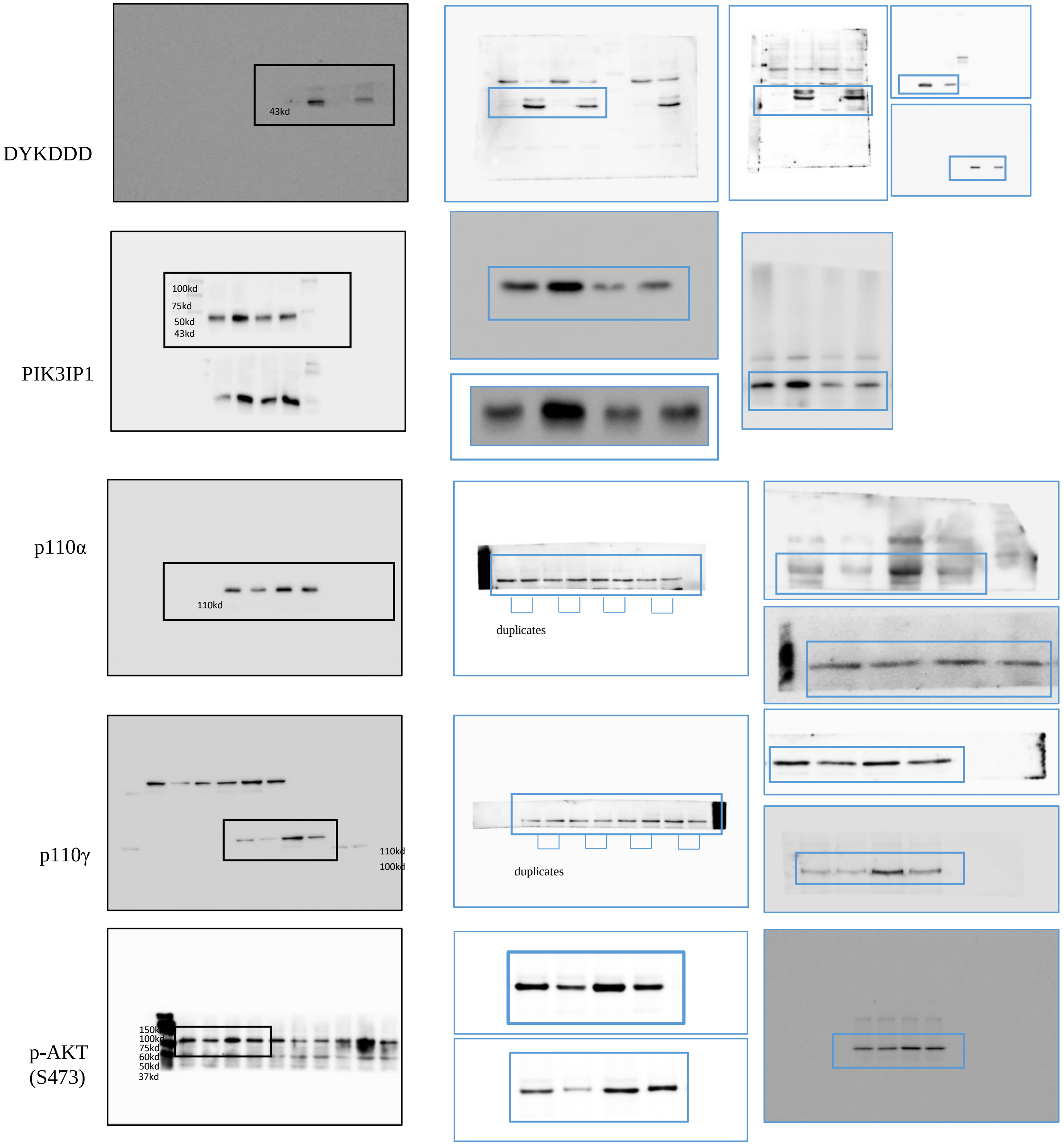

Supplementary Figure S5. Blots are for Figure 2A. Blots marked with black boxes are on the main figures and blots marked with blue boxes are replicates.

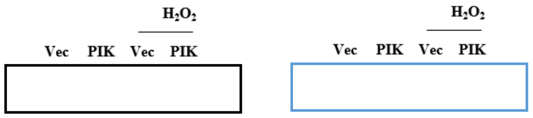

Supplementary Figure S6

Uncropped gel images for **Figure 2A**

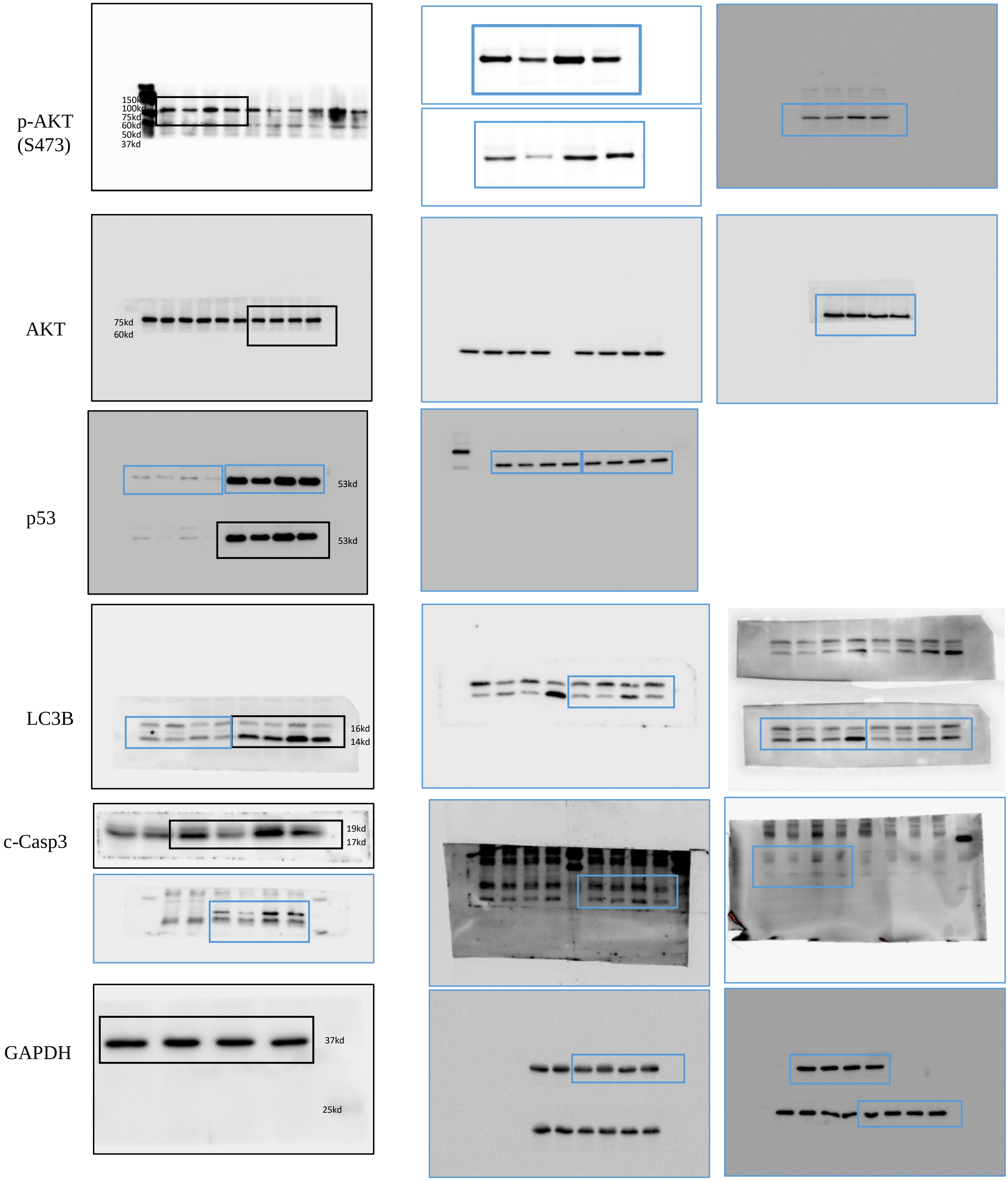

Supplementary Figure S6. Blots are for Figure 2A. Blots marked with black boxes are on the main figures and blots marked with blue boxes are replicates.

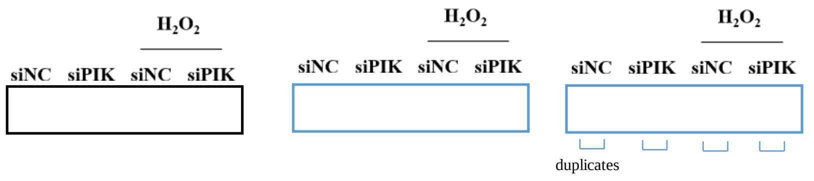

Supplementary Figure S7

Uncropped gel images for **Figure 3A**

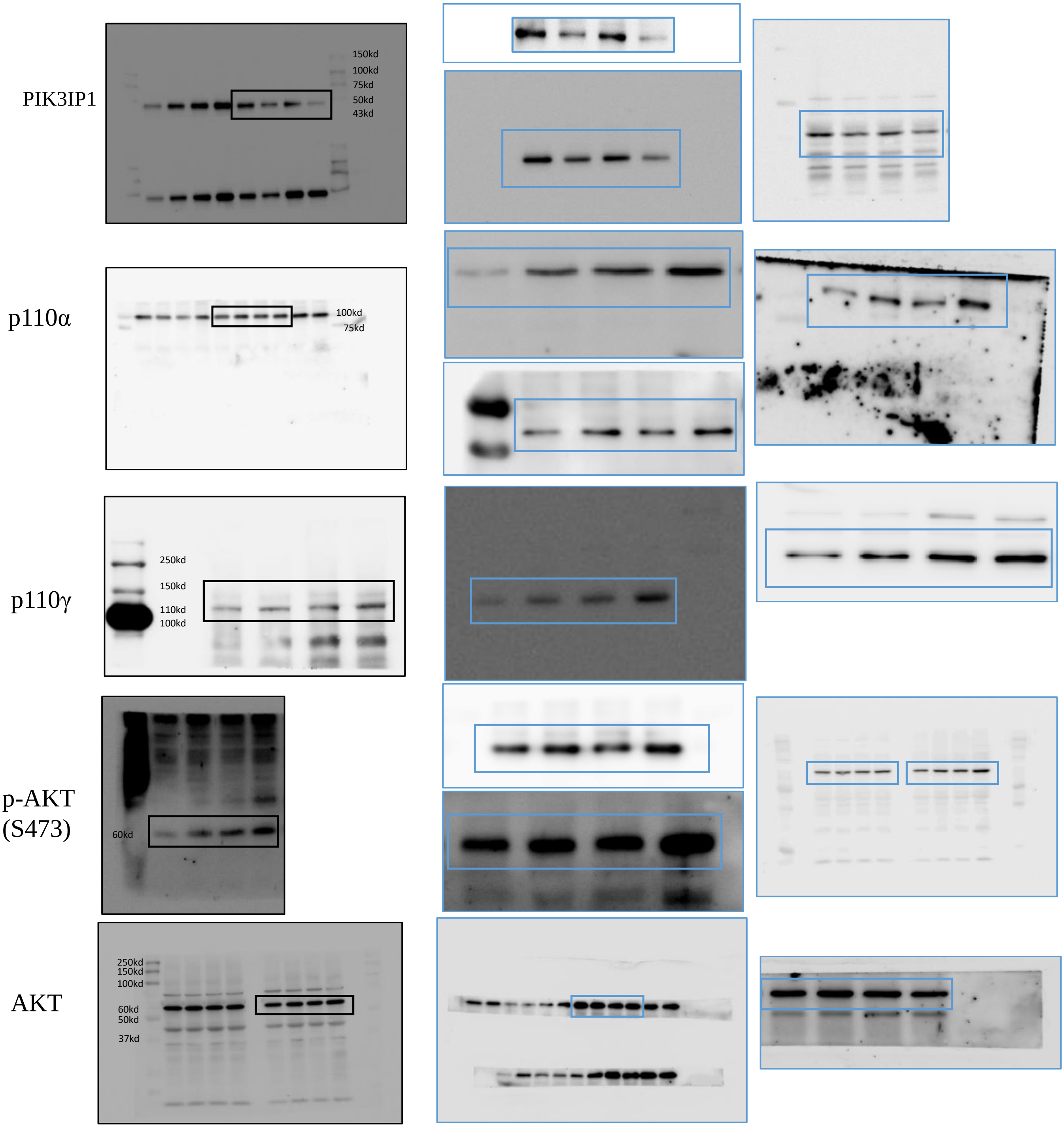

Supplementary Figure S7. Blots are for Figure 3A. Blots marked with black boxes are on the main figures and blots marked with blue boxes are replicates.

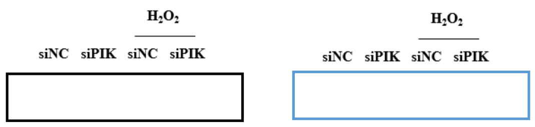

Supplementary Figure S8

Uncropped gel images for **Figure 3A**

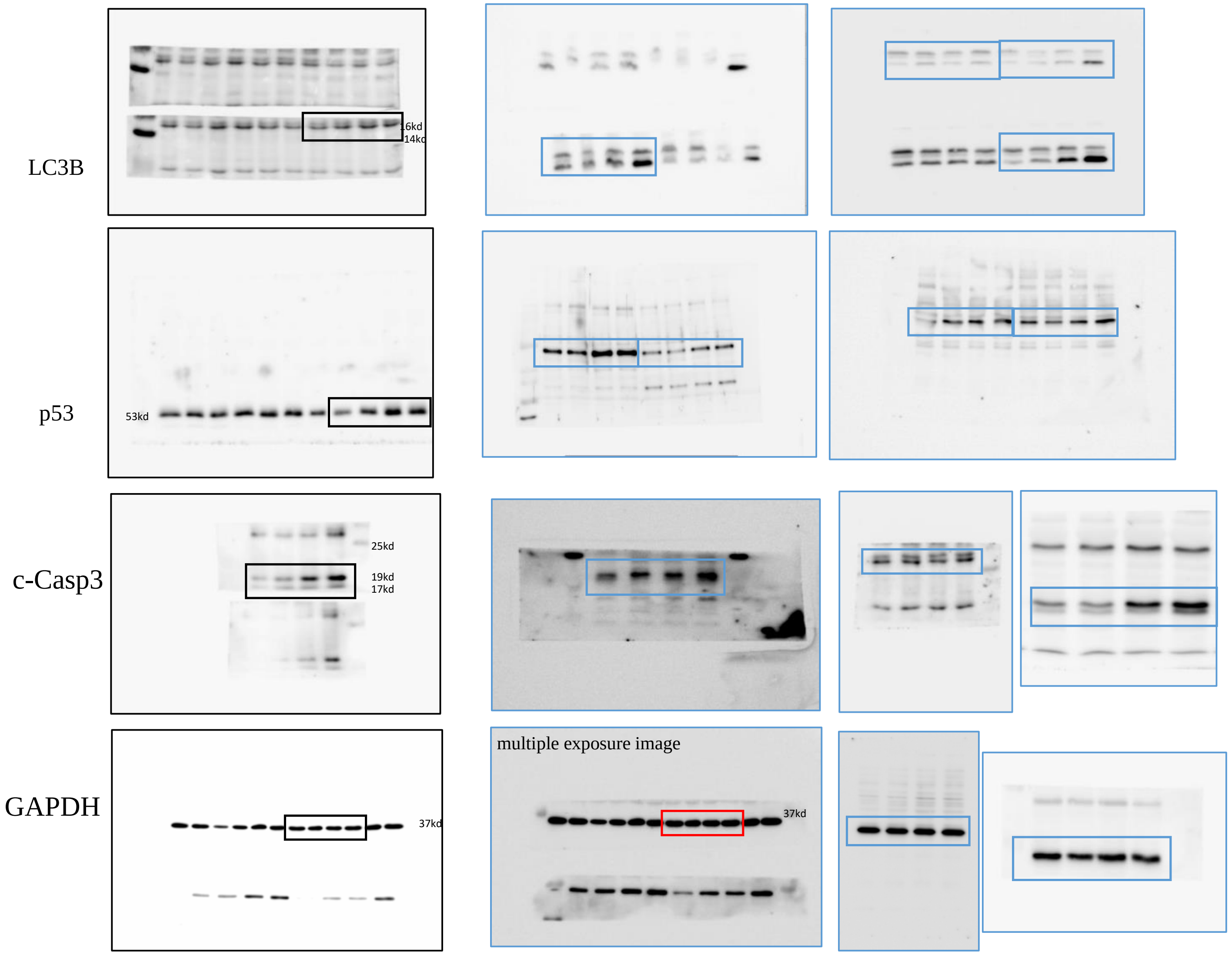

Supplementary Figure S8. Blots are for Figure 3A. Blots marked with black boxes are on the main figures and blots marked with blue boxes are replicates. GAPDH blots were overexposed. Blots marked with red boxes are multiple exposure images for GAPDH.

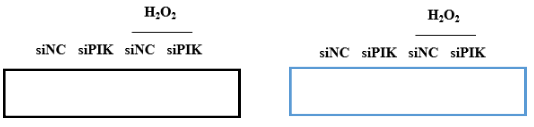

Supplementary Figure S9

Uncropped gel images for **Figure 4**

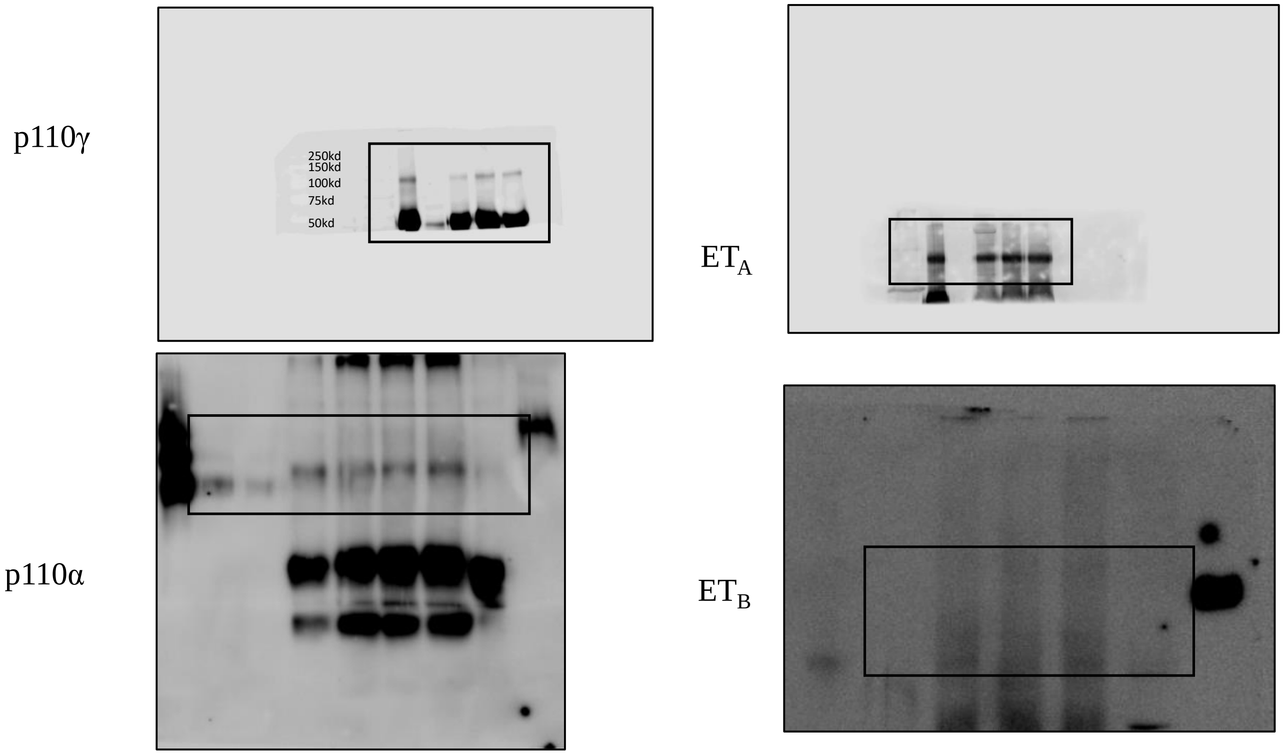

Supplementary Figure S9. Blots are for Figure 4. Blots marked with black boxes are on the main figures and blots marked with blue boxes are replicates.

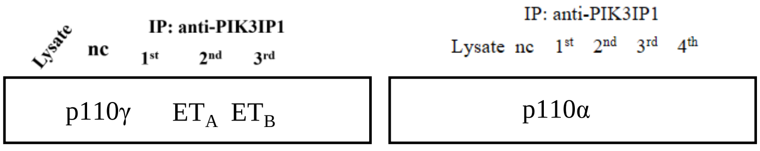

Supplementary Figure S10

Uncropped gel images for **Figure 5B**

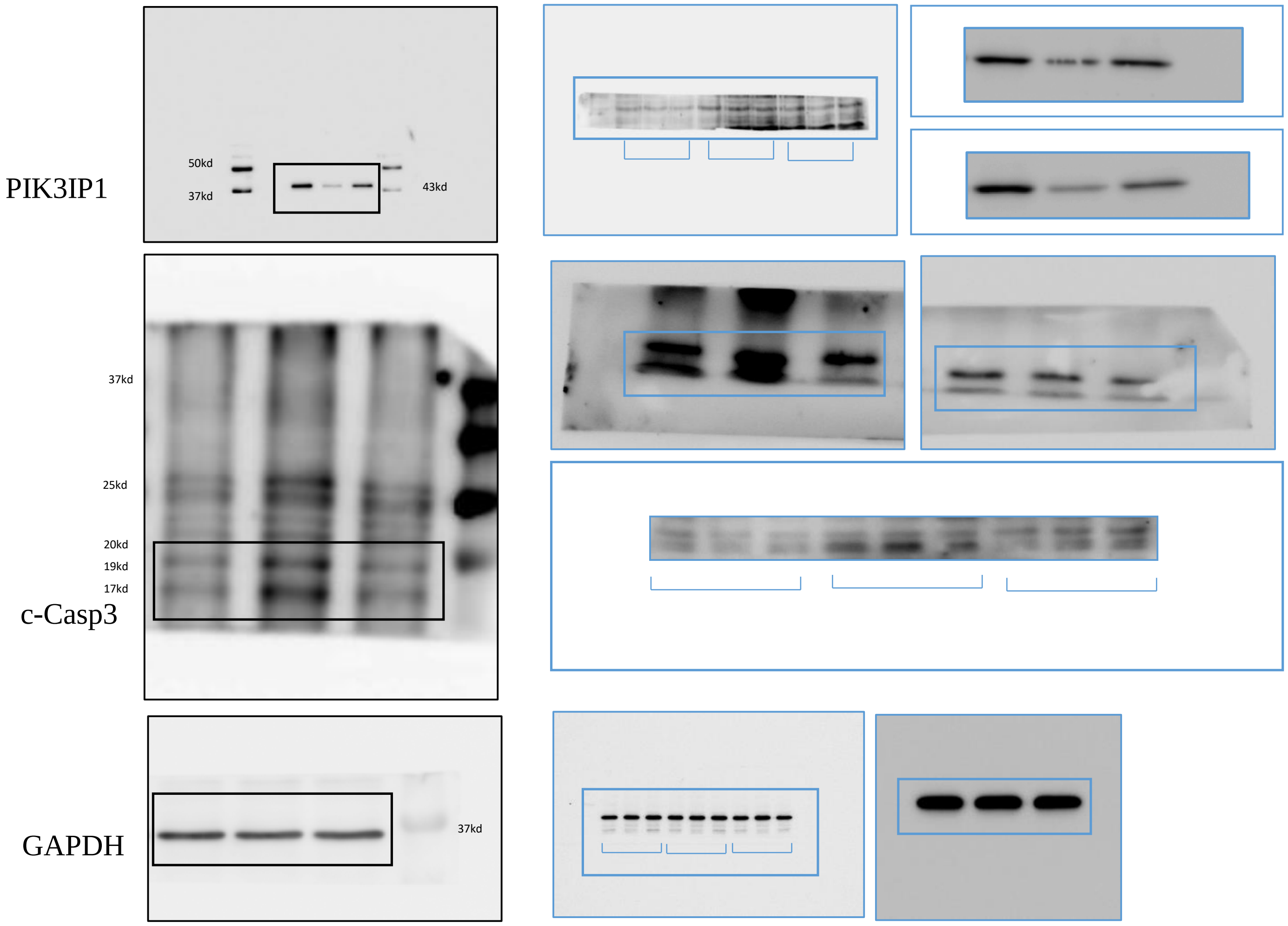

Supplementary Figure S10. Blots are for Figure 5B. Blots marked with black boxes are on the main figures and blots marked with blue boxes are replicates.

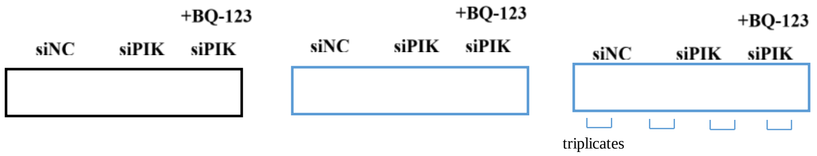

Supplementary Figure S11

Uncropped gel images for **Figure 6A**

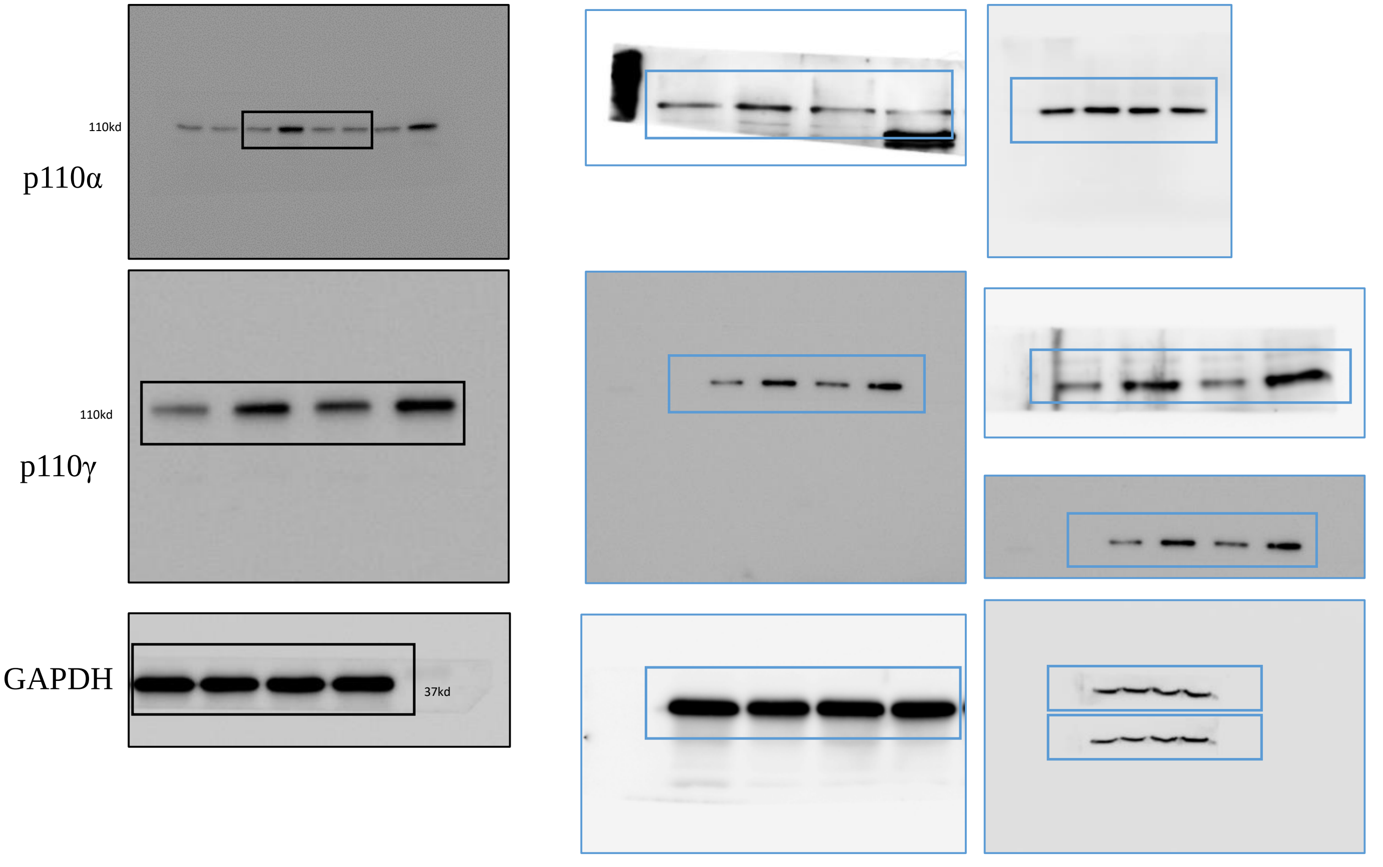

Supplementary Figure S11. Blots are for Figure 6A. Blots marked with black boxes are on the main figures and blots marked with blue boxes are replicates.

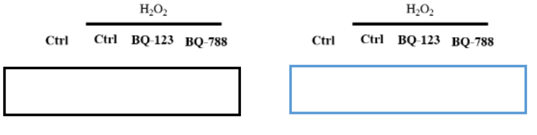

Supplementary Figure S12

Uncropped gel images for **Figure 6B**

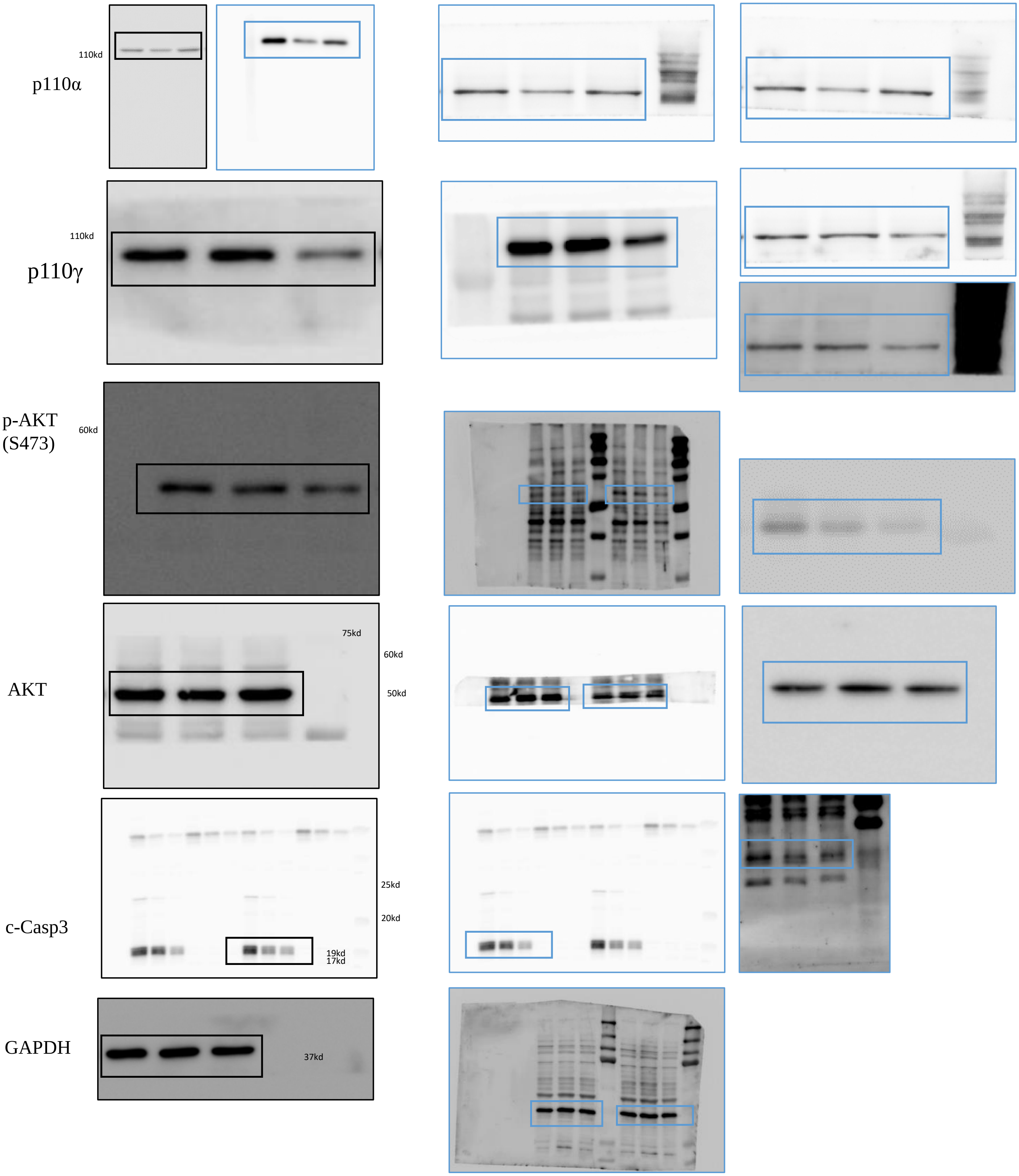

Supplementary Figure S12. Blots are for Figure 6B. Blots marked with black boxes are on the main figures and blots marked with blue boxes are replicates.

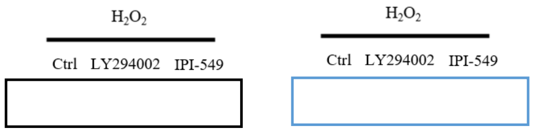

Supplementary Figure S13

Uncropped gel images for **Supplementary Figure S1C**

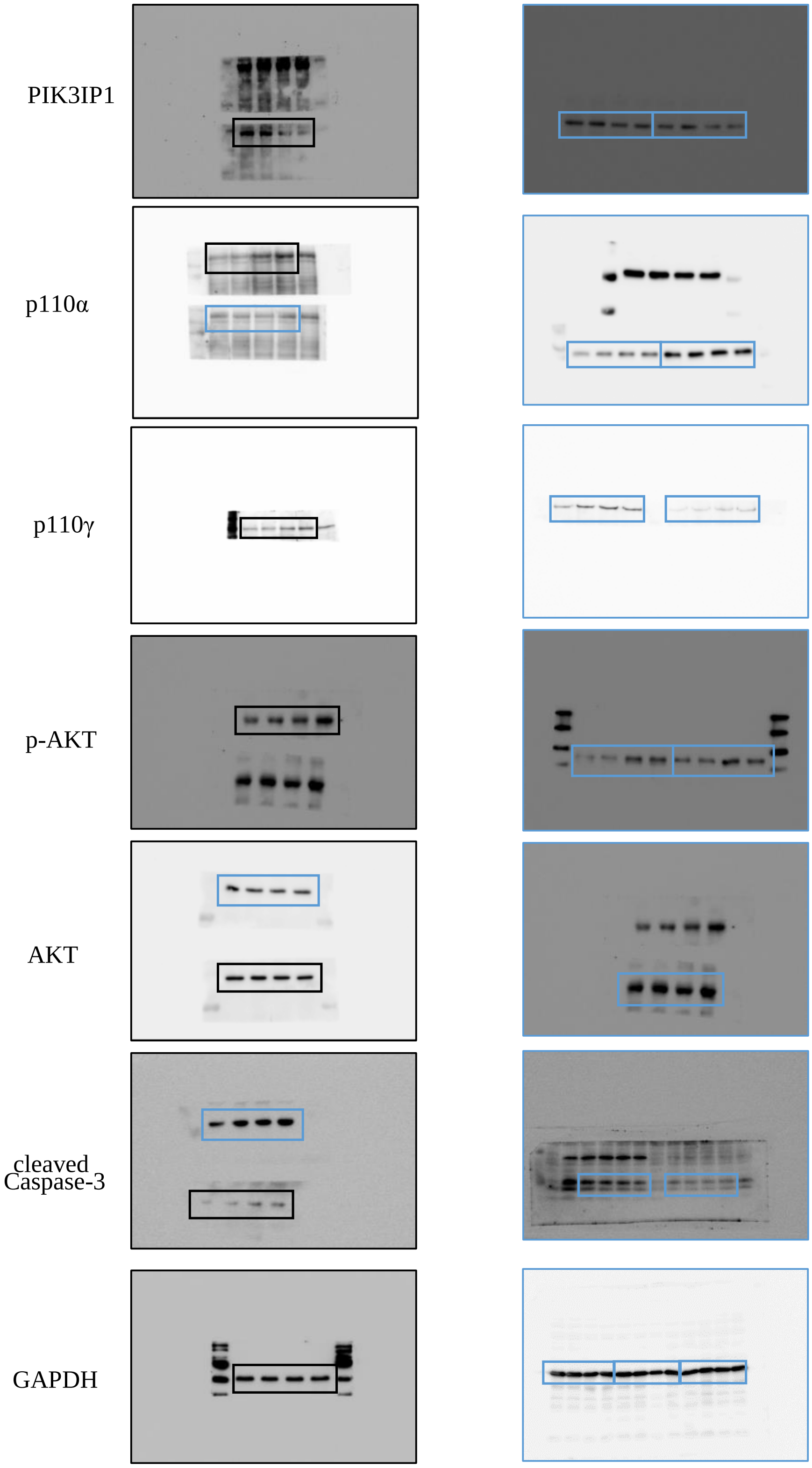

Supplementary Figure S13. Blots are for Supplementary figure 1C. Blots marked with black boxes are on the main figures and blots marked with blue boxes are replicates.

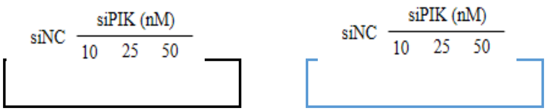

Supplementary Figure S14

Uncropped gel images for **Supplementary Figure S1F**

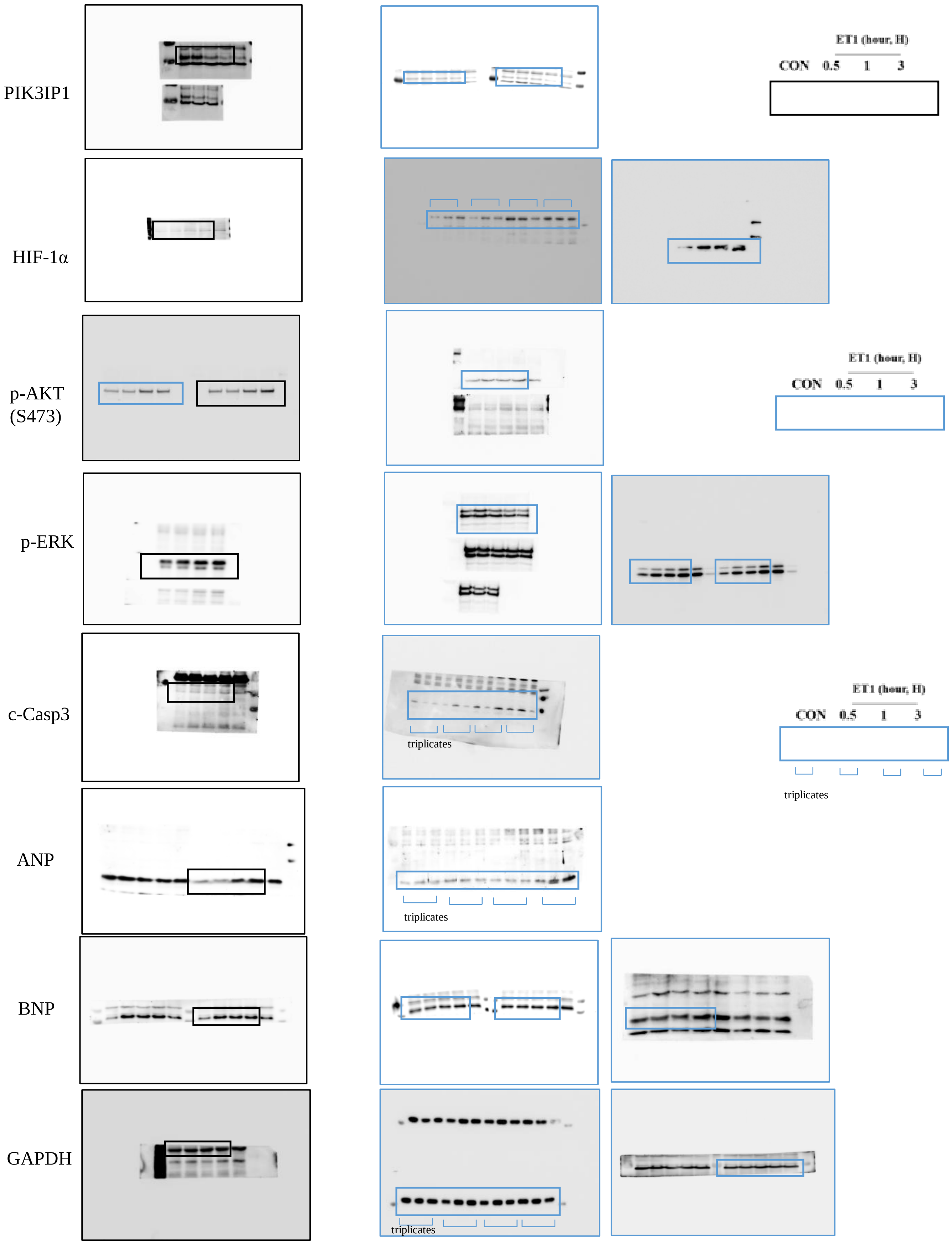

Supplementary Figure 14. Blots are for Supplementary figure 1F. Blots marked with black boxes are on the figures and blots marked with blue boxes are replicates.

Supplementary Figure S15

Uncropped gel images for **Supplementary Figure S2A**

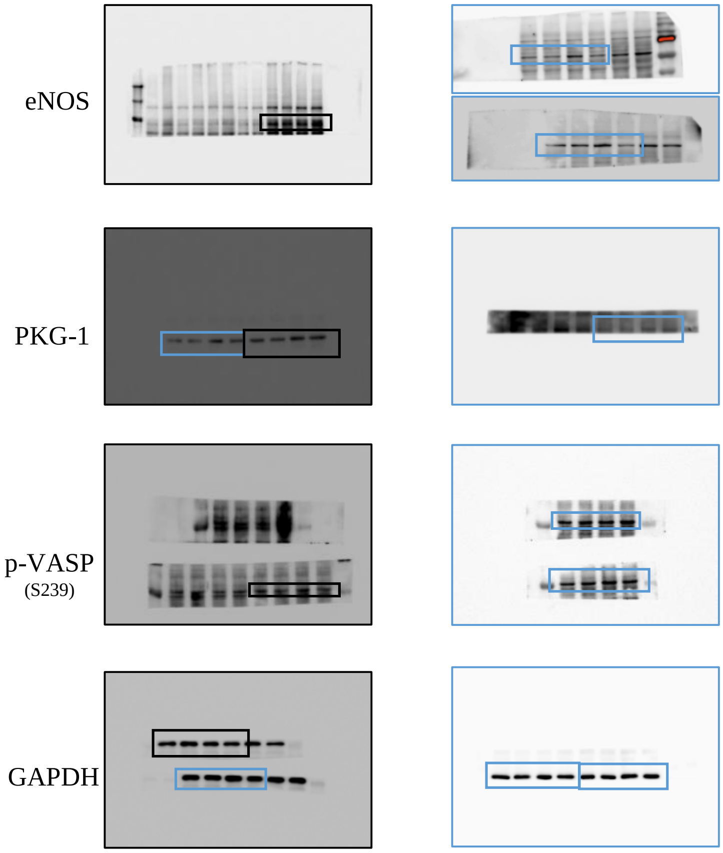

Supplementary Figure S15. Blots are for Supplementary figure 2A. Blots marked with black boxes are on the figures and blots mark with blue boxes are replicates.

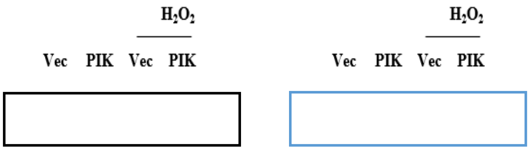

Supplementary Figure S16

Uncropped gel images for **Supplementary Figure S2C**

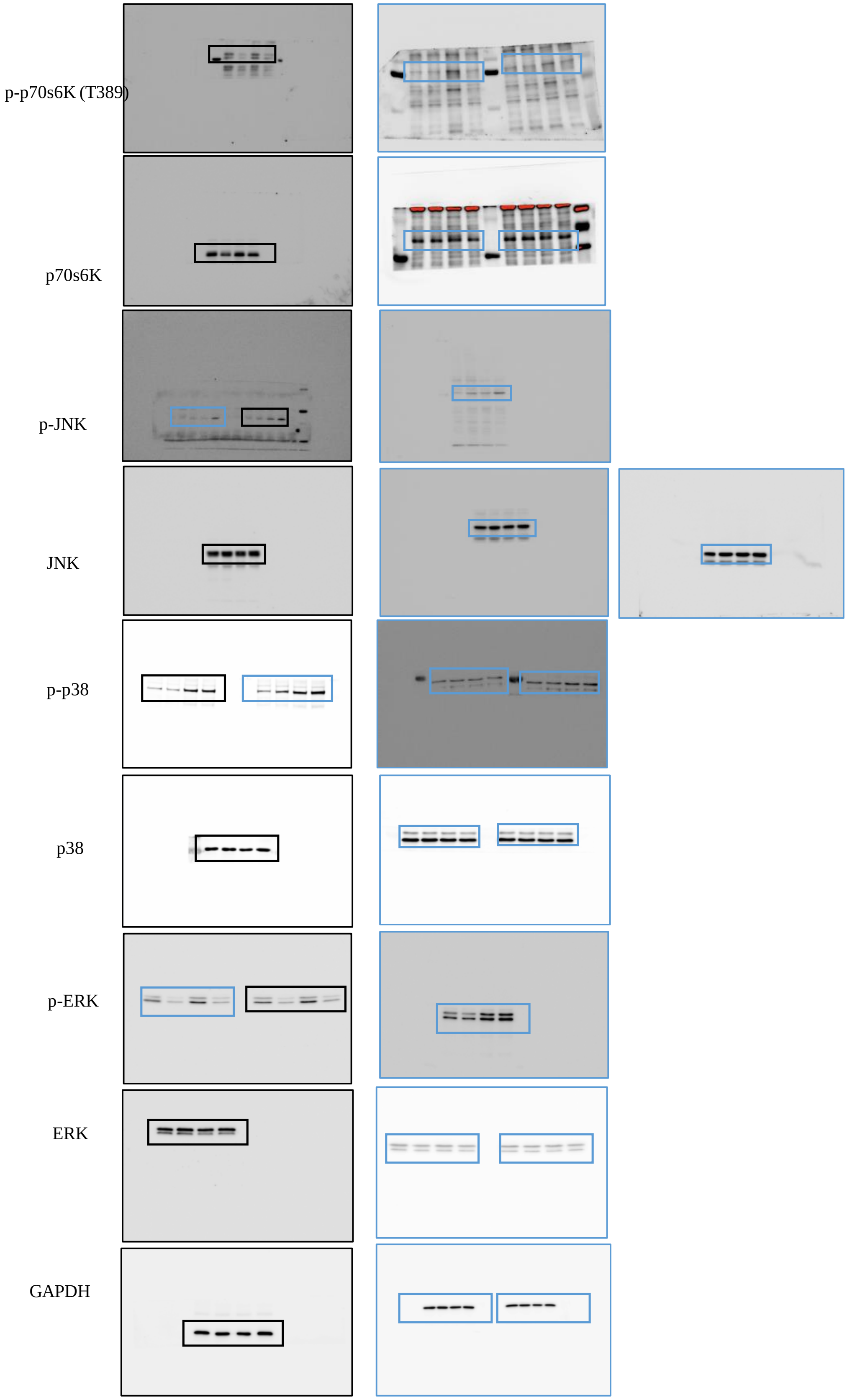

Supplementary S16. Blots are for Supplementary figure 2C. Blots marked with black boxes are on the figures and blots marked with blue boxes are replicates.

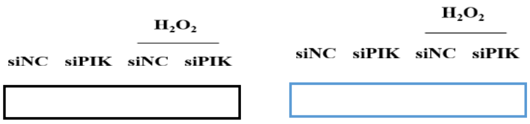

Supplement: Supplementary file 1 [file cells-11-02162-s001.zip › cells-1780958-supplementary.pdf]
